# Trends in the Contraceptive Method Mix in Low- and Middle-Income Countries: Analysis Using a New “Average Deviation” Measure

**DOI:** 10.9745/GHSP-D-14-00199

**Published:** 2015-03-02

**Authors:** John Ross, Jill Keesbury, Karen Hardee

**Affiliations:** a Futures Group, Washington, DC, USA; b Population Council, Washington, DC, USA

## Abstract

The method mix of contraceptive use is severely unbalanced in many countries, with over half of all use provided by just 1 or 2 methods. That tends to limit the range of user options and constrains the total prevalence of use, leading to unplanned pregnancies and births or abortions. Previous analyses of method mix distortions focused on countries where a single method accounted for more than half of all use (the 50% rule). We introduce a new measure that uses the average deviation (AD) of method shares around their own mean and apply that to a secondary analysis of method mix data for 8 contraceptive methods from 666 national surveys in 123 countries. A high AD value indicates a skewed method mix while a low AD value indicates a more uniform pattern across methods; the values can range from 0 to 21.9. Most AD values ranged from 6 to 19, with an interquartile range of 8.6 to 12.2. Using the AD measure, we identified 15 countries where the method mix has evolved from a distorted one to a better balanced one, with AD values declining, on average, by 35% over time. Countries show disparate paths in method gains and losses toward a balanced mix, but 4 patterns are suggested: (1) rise of one method partially offset by changes in other methods, (2) replacement of traditional with modern methods, (3) continued but declining domination by a single method, and (4) declines in dominant methods with increases in other methods toward a balanced mix. Regions differ markedly in their method mix profiles and preferences, raising the question of whether programmatic resources are best devoted to better provision of the well-accepted methods or to deploying neglected or new ones, or to a combination of both approaches.

## Abstract (precis)

Applying a standard measure of the method mix evenness suggests 4 patterns among 15 countries moving toward a more balanced mix: (1) rise of one previously underrepresented or new method, (2) replacement of traditional with modern methods, (3) continued but declining domination by a single method, and (4) general movement toward a balanced mix. Improving availability of underutilized or new methods can improve the method mix, although better implementation of more popular methods might increase contraceptive use more expeditiously.

## INTRODUCTION

It has long been recognized that the availability of only 1 or 2 contraceptive methods in a country constrains total contraceptive use and limits the options that women and couples have to manage their pregnancies. Conversely, adding methods expands choice for women and men and increases contraceptive use. With renewed attention to improving access to family planning services that respect and protect human rights, including access to a fuller, more informed choice of methods, experts have considered what it means for programs to offer a full range of contraceptive methods.^1^^,^^2^ In some countries, governments or markets have not enabled access to certain modern methods; in other countries, some methods are inherently unpopular, as with vasectomy and the condom. In some settings, such as rural Africa, certain methods are not easily accessible because they are clinically difficult to implement, as with sterilization or the intrauterine device (IUD). Clearly, the current contraceptive method mix is severely unbalanced in many countries, with over 50% of all use by a single method or with only 2 methods accounting for most use.^3^

In many countries, a single contraceptive method accounts for more than 50% of all contraceptive use.

Experiences with changes in the contraceptive method mix are of interest to policy makers and donors, since a broader mix expands contraceptive method choice, letting women and couples choose the method that suits them best and change methods as their circumstances and needs change. How to expand method mix remains an important programmatic question. This entails a focus mainly on the “share” of use held by each method—that is, the portion of all use, with the sum of the portions always adding to 100%. A totally balanced mix, with even shares for all methods, is never a program objective since it would mean, for example, that condom use would equal that of the implant and IUD use would equal that of male sterilization. Instead, the objective is to generally move away from an obviously distorted mix, without specifying precisely how fully balanced the mix should be, while enlarging access to a wider variety of method choices. In practice, it is easier to know when a mix is seriously distorted than it is to specify the ideal mix, due not only to program limitations but also to strong social norms that may block adoption of a particular method. Over time, countries will vary in which methods gain or lose shares, while total use in most cases will increase.

The aim is not to prescribe a specific ideal method mix but to generally increase access to a wider variety of method choices.

Exclusive attention to the contraceptive method mix, by itself, can give misleading results, since the same mix may prevail at both low and high levels of total use. A poor method mix can exist in a low-prevalence setting, such as Nigeria, but also in China, where the contraceptive prevalence rate (CPR) is in the eighties.^16^ Further, a rapid change in the method mix may distort the mix but simultaneously raise the overall level of use, as with the injectable in eastern and southern Africa. In that case, a more distorted method mix has actually increased choice by making an important method more available than before. In short, an analysis of mix changes over time also requires attention to changes in levels of total contraceptive use.

A poor method mix can prevail at both low and high levels of contraceptive use.

This paper reviews previous work devoted to measuring distortions in the mix, and it builds on that work by analyzing trends over time in the distribution of users across 8 contraceptive methods, using data available in national surveys in low- and middle-income countries (LMICs). The paper introduces a new measure of distortions in the method mix—the “average deviation” of method shares around their own mean. This measure is used in conjunction with total contraceptive use to give a more complete and programmatically useful picture of trends over time, along with possible programmatic responses to improving the method mix.

## PREVIOUS MEASURES OF METHOD MIX SKEW

A number of studies have focused on the “skew” in the method mix. A method mix is clearly distorted, or skewed, when a single method covers more than half of all use (the 50% rule). A series of 3 analyses has applied the 50% rule to large numbers of national surveys,^4^^–^^6^ and we build on this work below. The latest report found that 30% of 109 developing countries suffered from a skewed method mix,^4^ down from 35% in a 2006 analysis.^6^ Among the contraceptive methods, the injectable showed increases in use, while sterilization and the IUD showed relative declines.

Other studies have analyzed the use of specific contraceptive methods within the method mix. A United Nations (UN) report covering both developed and developing countries found that, globally, more than half of all users relied on either female sterilization or the IUD.^3^ Use was highly concentrated: in nearly every country, 1 or 2 methods accounted for over half of all use among married/in-union women. However, regions differed sharply in their particular method mixes. The UN found that little change occurred in the mix of methods between 1990 and 2011, either globally or within individual regions. Still, some increase occurred during that period for the injectable and some decline occurred for traditional methods. The pill had the widest geographic spread, and male methods the least; such imbalances limit easy movement between methods to adjust to personal circumstances and aims.

The increase in injectable use, noted in the UN review, has modified the method mix in eastern and southern African countries and elsewhere, as reviewed by Adetunji^7^ and by Sutherland et al.^8^ Ross and Agwanda^9^ showed that the injectable increase has been mainly additive to the prior level of total use, rather than substituting for other methods. While its popularity has raised total use in many settings, it is a short-acting method, and questions still remain about the frequency of its discontinuation and switching to other methods. Little is known about which methods, if any, women turn to when they discontinue use; in a review of 23 countries, Ali and Cleland^10^ found that among users of modern methods who discontinued, as of 3 months later, 26% were at risk of becoming pregnant (i.e., they were not using any method), 10% were pregnant, and 60% had switched to another method (median values).

Increased injectable use has been mainly additive to prior levels of contraceptive use, rather than substituting for other methods.

When the current mix is augmented by access to an additional method, total use tends to rise. Asian examples were noted by Freedman and Berelson^11^ and by Jain,^12^ who estimated for Taiwan that adding 1 method to the mix would increase total use by about 12 percentage points (e.g., from 30% to 42% total contraceptive prevalence). Ross and Stover^13^ estimated that making 1 additional method available to at least half of the population raised modern contraceptive use by 4–8 percentage points, based on 27 years of data for 113 countries.

Adding more methods to the method mix tends to increase total contraceptive use.

Some of the literature on method mix has occasionally been directed to specifying method mixes that are most appropriate for different user profiles and life objectives^14^ or to tools that relate the individual needs of women to the characteristics of alternative contraceptive methods.^15^

## DATA AND METHODS

The primary set of national surveys used in our analysis is a thorough compilation provided by the UN Population Division of some 700 surveys from both developed and developing countries.^16^ After exclusions to remove surveys from developed nations, including eastern Europe, and those that lack breakdowns by individual method, 666 surveys from 123 countries were included in this analysis (Appendix 1). Most tabulations are for the most recent surveys for the 123 countries.

This analysis draws on data from 666 national surveys in 123 countries.

Regarding the latest surveys in these 123 countries, most were of 3 types: over 40% were from the Demographic and Health Surveys (DHS), about a fourth were from the Multiple Indicator Cluster Survey (MICS) series, another fourth from independent national surveys, and the rest from other sources. By region, 38% were for sub-Saharan Africa, 18% each for Asia and North Africa/West Asia, 21% for Latin America, and 4% for Central Asia. Regarding timing, 80% of surveys were conducted from 2005 onward. The percentages of surveys in each 5-year period starting in 1995 were 5% (1995–1999), 15% (2000–2004), and 57% (2005–2009), with an additional 23% between 2010 and 2012.

The surveys provide the percentage of married women using the 8 contraceptive methods (i.e., contraceptive prevalence) that together account for most use and for which data are available for most countries. “Modern” methods include male and female sterilization, the IUD, oral contraceptive pills, the injectable, the condom, and the implant. Traditional methods are composed mainly of withdrawal and rhythm, in equal parts, representing 92% of all traditional method use (average across all DHS surveys). We could have performed separate analyses on small sets of countries where other methods were used by non-trivial percentages of women, but the aim of detecting marked changes in the overall mix in most countries required attention to the 8 methods studied here.

We converted contraceptive prevalence rates provided in the surveys (with all married women as the denominator) to the percentage of use due to each contraceptive method, totaling 100% (with users of any contraceptive method as the denominator). Changes in these latter percentages over time made up the focus of this study. Where regional or other averages (means) are given, they are unweighted, so every country receives the same importance in the averages. The patterns would be somewhat different if they were weighted by population size. Certain individual countries are selected for separate discussion, and country details appear in several tables.

### The Average Deviation (AD) Measure

Countries differ in their method mixes, all falling between the possible extremes of reliance on a single method to reliance on all methods equally; moreover, mixes change over time. How large must a change be to signal an important shift? Small shifts in surveys can come from noise in the data and/or from sampling error, while very large shifts usually reflect real and important changes in behavior.

Therefore, a standard measure of the evenness of the method mix is needed, to gauge the extent of change and to compare one country or region with another. After a review of possible measures, we chose to use the “average deviation” (AD); it is the simple average of the absolute differences around the mean (average), whether positive or negative, and for our purposes is preferable to the standard deviation, which squares deviations, including those of extreme outliers.

The average deviation provides a measure of the evenness of a country's method mix and allows for comparisons across countries.

If all 8 methods held equal shares, they would each have 12.5% of all users (100% divided by 8), and 12.5% is always the average value. In every country, the share of each method in the mix varies around that average. Almost always, the implant, male sterilization, and the condom fall below the average. Depending on the region and country, any of the other methods (the pill, injectable, IUD, female sterilization, or traditional methods) can rank either above or below the average, e.g., female sterilization ranks below the average in sub-Saharan Africa and the IUD ranks above the average in the Middle East.

The AD measure captures the disparity in the mix, i.e., the sum of the 8 absolute differences around the average of 12.5%. The sum of those differences can range from 0 to 21.9: if every method is used equally, no differences exist and the sum is 0, and if a single method covers all use, the differences sum to 21.9. Most AD values range from 6 to 19; across all surveys, the interquartile range (i.e., the middle 50% of values, between the 25th and 75th percentiles) is from 8.6 to 12.2.

Thus, a high AD value indicates a skewed method mix, with dominance of a few methods, and a low AD value indicates a more uniform pattern across the methods. No country has a fully uniform mix with an AD value of 0, and as noted above, perfectly even shares would not likely be a program objective. In some cases, a rapid movement toward a new method might be sought programmatically, disturbing a relatively even mix.

A high average deviation value indicates a skewed method mix while a low value indicates a more uniform pattern across methods.

The relationship between the mix and the total CPR must be kept in mind, as a low AD can occur at any level of the CPR. For example, in Nigeria the CPR is low whereas in Peru the CPR is high, and both have low AD scores.

The AD can also be calculated for the 7 modern contraceptive methods, the ADM; however, the dynamics of changes in contraceptive method mixes depend upon the competition between modern methods and traditional methods, usually with the former partially replacing the latter, so the AD measure is preferred.

We also consider an alternative measure of method mix skew, since this work builds upon the publications described above that employ the “50% rule” as the measure of the mix's imbalance, and the results are compared below with those from the AD measure.

## FINDINGS

### Variations on the 50% Rule to Define Skew

The 50% rule tells whether a single contraceptive method accounts for more than half of all use. That has been a useful measure, and it is of interest to assess how sensitive the results are to exactly 50%. It is possible to vary it, to examine changes in the outlook according to the severity of the rule. If a single method must cover 60% of all use for skew to occur, fewer countries will qualify, and if the rule is 40%, more will qualify.

The 50% rule, commonly used to measure method skew, identifies countries where a single method accounts for more than half of all use.

Table 1 presents the counts according to a range of cutoff rules, from 30% to 70%, first for the 123 countries using only the latest surveys, and second for all 666 surveys to encompass some of the historical experience. By the 50% rule, 28% of countries and 34% of surveys qualify as having a skewed method mix, a finding close to that of Bertrand et al.,^4^ which is expected since the 2 sets of data contain many of the same countries. Both results suggest some improvement: the higher figures for all surveys reflect more skew in the past, which agrees with the finding by Bertrand et al. of a decline from 35% to 30% of countries that are skewed.

**Table 1.** Percentage of Countries (Latest Surveys) and of All Surveys With Method Mix Skew According to the Cutoff Level^a^

	**No. (%)**	
**Cutoff Level**	**Countries (Latest Surveys)**	**All Surveys**
30%	115 (94.3)	609 (91.4)
40%	68 (55.7)	393 (59.0)
50%	35 (27.9)	225 (33.8)
60%	22 (18.0)	137 (20.6)
70%	10 (8.2)	62 (9.3)

a The “50% rule” is the most commonly used cutoff level, indicating that a single contraceptive method accounts for more than half of all contraceptive use in a given country. Changing the cutoff level changes the severity of the rule.

By the 50% rule, 28% of countries have a skewed method mix.

Under the 40% rule, the skewness counts jump to over half (56%) of all countries and nearly three-fifths (59%) of all surveys. In the other direction, by the 60% rule, about a fifth of countries are still skewed (18% of countries and 21% of surveys), which still indicates major imbalance among methods. For particular countries (not shown), the rules matter: Kenya and Uganda, each with a maximum of 47% use with a single contraceptive method, would be included under the 40% rule but not under the 50% rule. Tanzania would qualify only under the 30% rule. On the other hand, some countries that qualify by the 50% rule would not qualify with the 60% rule. Examples in sub-Saharan Africa are Malawi (56%), Niger (56%), and Rwanda (51%), and elsewhere, Egypt (59%) and Mexico (55%). Regardless of the rule used to measure skew, the results show major shortfalls in making a wide variety of contraceptive choices available.

**Figure 1.**
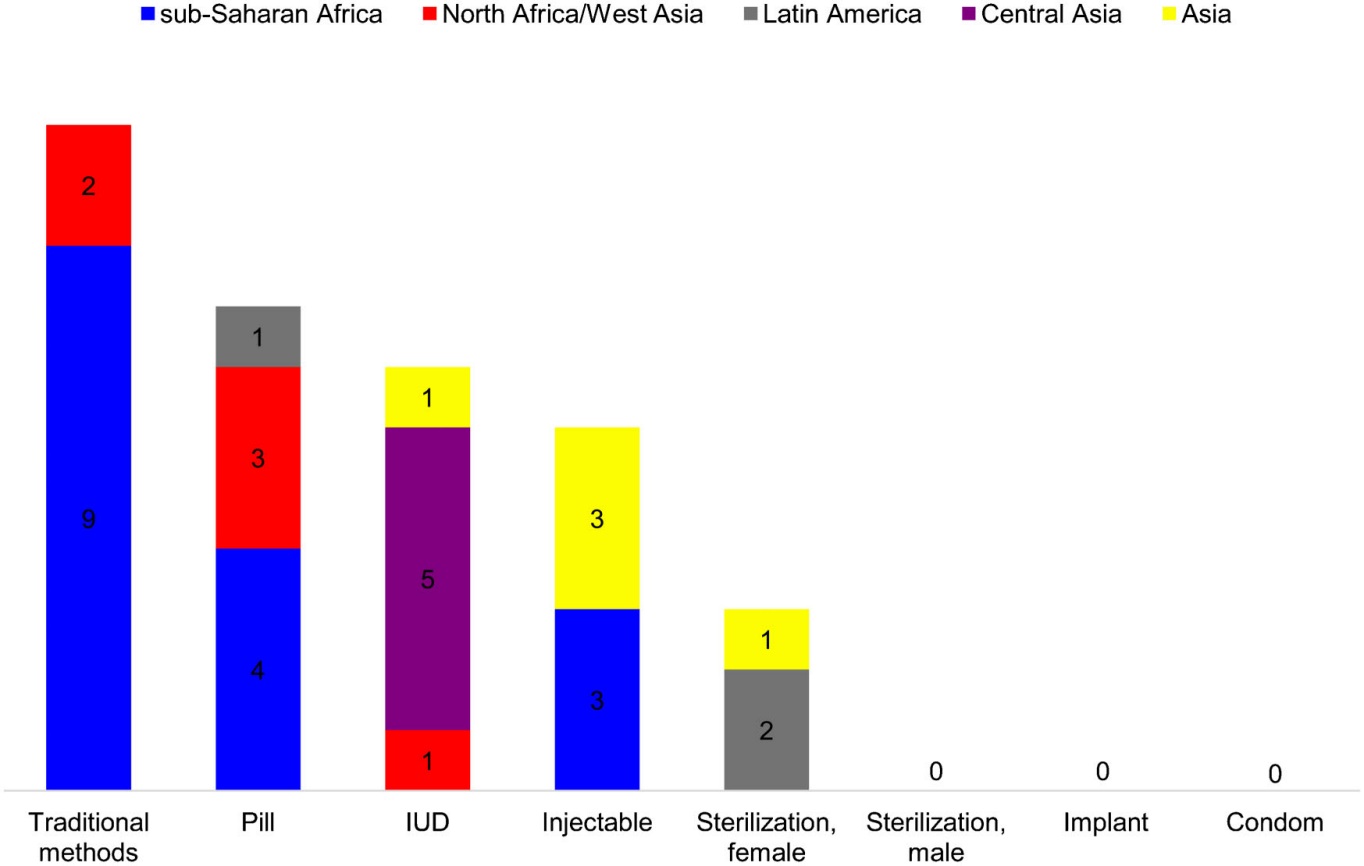
Number of Countries With Method Mix Skew According to the 50% Rule,^a^ by Dominant Method (N = 35 Countries) Abbreviation: IUD, intrauterine device. ^a^ Each method comprises over 50% of total contraceptive use in the country. Missing regions in the bars had no country with over 50% use of that method among contraceptive users; male sterilization, the implant, and the condom had no countries at all with over 50% use of those methods among contraceptive users.

The dominant method in countries with skewed method mix (by the 50% rule) is quite different by region (Figure 1). Traditional methods stand out in sub-Saharan Africa and to a lesser extent in North Africa/West Asia, and the pill is also popular in these 2 regions. The injectable is prominent in sub-Saharan Africa as well as in Asia. The IUD is important, but 5 of its 7 countries are the 5 Central Asia Republics. Female sterilization is dominant in 2 Latin American countries and 1 Asian country. The other 3 methods (male sterilization, the implant, and the condom) do not show skew anywhere. The figure is based on 35 countries, and for each country meeting the 50% rule, there are others in the same region just below 50%.

### Method Mix at the Global, Regional, and Country Levels

**Figure 2.**
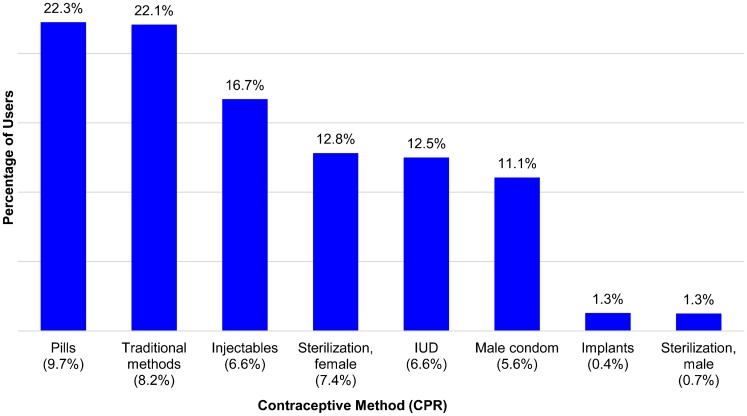
Method Mix Among Contraceptive Users Based on Latest Surveys for 123 Countries Abbreviation: CPR, contraceptive prevalence rate; IUD, intrauterine device.

The overall pattern of contraceptive use by method appears in Figure 2, for married/in-union women using any method across the 123 countries (all methods sum to 100% of users). Among these contraceptive users, 22% use the pill and another 22% use traditional methods, but the levels are best understood with attention to the contraceptive prevalence among *all* married/in-union women by method. Those percentages are much lower, and are given along the x-axis with the method labels. Among all users, 61% rely either on the pill, traditional methods, or the injectable, but that represents only 24.5% of married/in-union women.

61% of users rely on the pill, traditional methods, or the injectable, which represents about 25% of all married women.

Regions differ greatly from these averages, as shown in Table 2. Many contraceptive users (lower panel) in sub-Saharan Africa rely on traditional methods while fewer do so in Central Asia, where the injectable is nearly ignored and the IUD is paramount. Male sterilization plays a minor role in each region, while female sterilization varies from 2% to 28% of users, with high values only in Latin America and Asia. The injectable ranges widely, from 2% in Central Asia and up to one-fourth of all users in sub-Saharan Africa. The condom varies less by region, and accounts for around 11% of users overall. The implant is nearly negligible, but its use has increased recently in some countries.

**Table 2.** Contraceptive Prevalence Among Married/In-Union Women and Method Mix Among Contraceptive Users, Based on Latest Surveys in 123 Countries

****	**Any Method**	**Any Modern Method**	**Sterilization, Female**	**Sterilization, Male**	**Pills**	**Injectables**	**IUD**	**Male Condom**	**Implants**	**Traditional Methods**
**Prevalence Among Married/In-Union Women**										
All Regions	45.1	36.9	7.4	0.7	9.7	6.6	6.6	5.6	0.4	8.2
sub-Saharan Africa	27.2	21.3	2.0	0.1	6.4	7.3	0.9	3.2	0.7	6.1
North Africa & West Asia	52.6	36.5	3.5	0.1	14.2	1.6	12.1	4.5	0.1	16.2
Latin America	65.0	57.9	18.9	0.9	13.6	8.0	6.9	9.4	0.3	7.1
Central Asia	52.5	46.1	1.1	0.0	3.6	1.2	36.6	3.2	0.0	6.4
Asia & Pacific	52.3	44.8	9.9	2.0	9.5	8.9	8.2	5.3	0.4	7.5
**Method Mix Among Contraceptive Users**										
All Regions			12.8	1.3	22.3	16.7	12.9	10.7	1.3	22.1
sub-Saharan Africa			5.2	0.9	24.1	24.1	4.4	10.4	2.6	28.4
North Africa & West Asia			6.8	0.2	27.8	3.8	22.4	8.3	0.1	30.6
Latin America			27.9	1.2	21.3	13.0	10.3	14.5	0.5	11.3
Central Asia			2.0	0.0	7.3	2.2	70.1	6.4	0.0	11.9
Asia & Pacific			19.3	3.2	18.5	19.6	13.0	9.9	0.7	15.8

All data are presented as percentages.

The actual contraceptive prevalence appears in the upper panel of Table 2, which provides the percentage of all married/in-union women using each method. As noted, a method with a high percentage of users commonly reflects a much lower percentage of all married/in-union women.

Among individual countries (data not shown), for modern methods, the IUD is most often the dominant method, for example, in all 5 Central Asian Republics, in Viet Nam, in Egypt, and in some other Middle Eastern countries. Female sterilization ranks first in Brazil, the Dominican Republic, El Salvador, Haiti, and India. The pill is first in Bangladesh, Niger, and Zimbabwe. The injectable is first in Ethiopia, Indonesia, Malawi, and Rwanda. Vasectomy, the condom, and the implant are never the most commonly used method. Apart from modern methods, the most common maximum is for traditional methods, usually in countries with a low CPR, many of which are in sub-Saharan Africa.

### Method Mix by Age, Residence, and Wealth Quintiles

**Figure 3.**
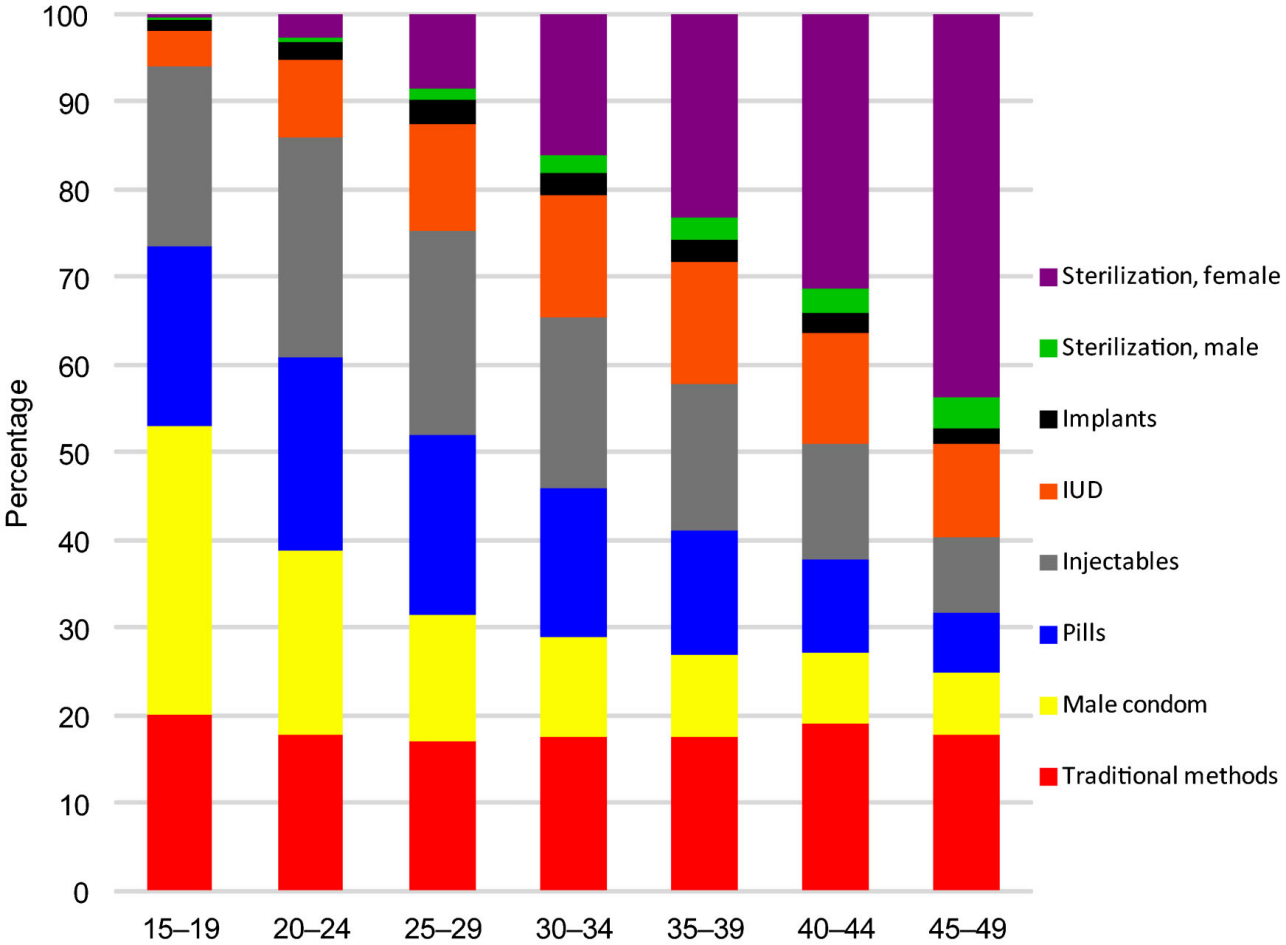
Method Mix Among Contraceptive Users by Age Abbreviation: IUD, intrauterine device.

The DHS set permits analysis of contraceptive use by personal characteristics, including age, residence, and wealth quintiles. The method mix changes systematically as women age (Figure 3). As age advances, the most notable growth is for female sterilization; its share is quite low below age 25 but then increases sharply and peaks at ages 45–49. All age groups neglect male sterilization and the implant. These results are for all regions merged; there is decidedly more selectivity by region and country. (For unmarried, sexually active adolescents, a clear shift occurs as youth age and gain more experience with contraceptive use. Between ages 15–19 and 20–24, reliance on the pill and injectable increases at the expense of the condom; data not shown.)

Method mix changes systematically as women age, with the most notable growth for female sterilization.

By residence, rural users show somewhat more use of traditional methods and injectables than urban users do, and less use of the condom (data not shown). However, the differences are relatively small. Total contraceptive prevalence averages 47.7% and 36.1% for urban and rural areas, respectively.

According to wealth quintiles, method shares are not greatly different (data not shown). While the quintiles differ sharply in total contraceptive prevalence (from 29.9% to 34.5% to 38.0% to 42.2% and 49.9% in sequence across the 5 quintiles), the profiles of their method preferences are basically similar. The main exception is the greater reliance on traditional methods by the bottom quintile as well as somewhat greater reliance on the injectable.

### Dynamics of Method Mix Changes Using the Average Deviation Measure

The method mix in some countries has remained skewed over time, while in others it has changed substantially. Of particular interest are 15 countries with marked improvements to their method mixes, which show patterns that may suggest potential program actions for other countries wishing to adjust the mix.

To identify these illustrative countries, the AD values for each country were themselves assessed for their degree of variation and for the degree of linearity for the decline in the values. That isolated countries that had experienced a historic reduction and regular decline in skew. In more detail, first a statistical measure of variability was applied to the full series of AD values in each country. In many countries, variability was small, indicating little change over time. In others, the changes were erratic. Leaving those aside, the countries with large AD variability were examined to identify those with a linear pattern of decline in the values, suggesting a historic regularity in the changes. The 15 cases here represent countries where the method mix has changed considerably and fairly regularly over time, as holding the greatest interest for programmatic strategies to reduce extreme skews in the mix.

**Figure 4.**
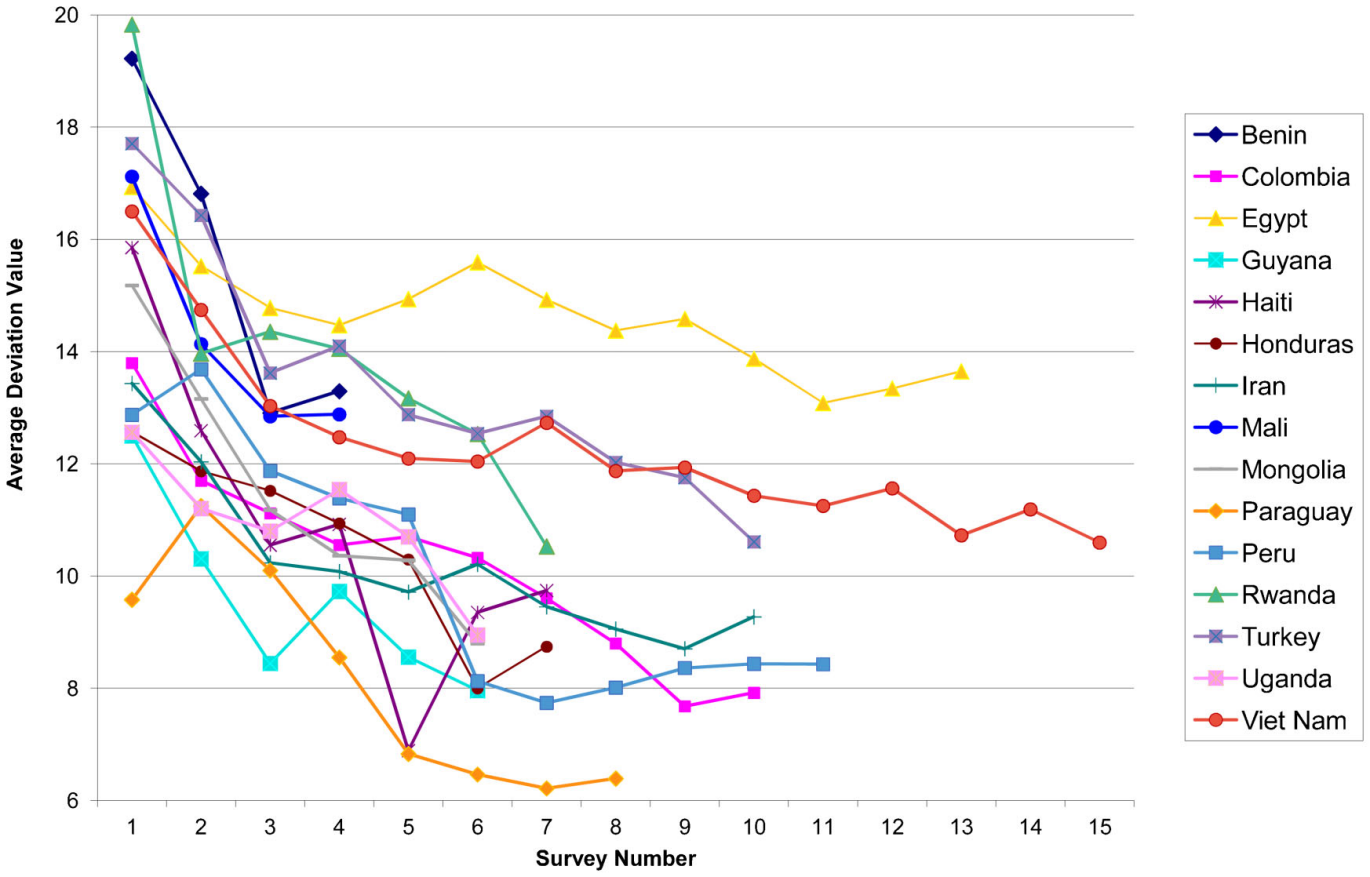
Declining Trends in Method Mix Skew Based on Average Deviation Values in 15 Selected Countries

Downtrends in the AD values are shown in Figure 4, using the full sequence of available surveys for each country. For each line in the figure, survey number 1 represents the earliest survey conducted in that country, and each subsequent survey follows. (The dates for the data points differ among the countries and cannot be displayed separately; the time intervals also vary between surveys. See Appendix 2 for all survey dates and details on method use.) The longest series shown are 15 for Viet Nam, starting in 1988, and 13 for Egypt, starting in 1974–75.

In Figure 4, the AD values are highest at the left, at nearly 20 for Rwanda and Benin, for early dates when traditional methods dominated the mix and the CPRs were quite low. On the other hand, at the right, low AD values are shown for 3 countries clustered near the bottom, for Iran, Peru, and Colombia. In all 3 countries, traditional methods were very dominant at the start, fading steadily over the years. However, the 3 countries took quite different paths for which methods gained in the method mix share and which ones lost, as discussed below. In Iran, the pill declined in share as use of the IUD and female sterilization gained. Peru illustrates the opposite, where the IUD and female sterilization declined while the injectable and condom came into better balance with them. In Colombia, the trends were very regular over the years, with declines from high early values for the IUD and pill, while female sterilization, the injectable, and condom gained. Note that the shares for sterilization in all countries come from adoptions during numerous past years, unlike shares for resupply methods, which reflect behavior more recently and have shares that are more sensitive to program changes year by year.

Reflecting the long-term transitions toward a more even mix in these 15 countries, the changes in the AD from the initial to the latest surveys have been substantial, down by 5.3 points, on average, for a 35% decline (Table 3).

**Table 3.** Average Deviation (AD) Values for Method Mix Skew in Initial and Latest Surveys, 15 Countries

**Country, Initial & Latest Survey Year**	**AD Value in Initial Survey**	**AD Value in Latest Survey**	**Decline**
**sub-Saharan Africa**			
Benin, 1981/82 & 2006	19.2	13.3	5.9
Mali, 1987 & 2006	17.1	12.9	4.2
Rwanda, 1983 & 2010/11	19.8	10.5	9.3
Uganda, 1988/89 & 2011	12.6	8.9	3.7
**North Africa & West Asia**			
Egypt, 1974/75 & 2008	16.9	13.7	3.2
Iran, 1976/77 & 2002	13.4	9.3	4.1
Turkey, 1963 & 2008	17.7	10.6	7.1
**Asia**			
Mongolia, 1994 & 2008	15.2	8.8	6.4
Viet Nam, 1988 & 2010/11	16.5	10.6	5.9
**Latin America**			
Colombia, 1969 & 2010	13.8	7.9	5.9
Guyana, 1975 & 2009	12.5	8.0	4.5
Haiti, 1977 & 2005/06	15.9	9.7	6.2
Honduras, 1981 & 2005/06	12.6	8.7	3.9
Paraguay, 1977 & 2008	11.2	6.4	4.8
Peru, 1969/70 & 2010	12.9	8.4	4.5
**Means**	15.2	9.8	5.3

Average deviation in the method mix of 15 countries declined, on average, by 35% over time.

The overall CPR levels vary considerably among these countries, since the same AD value can occur at either a high or a low level of overall contraceptive use. However, there is evidence that lower AD values, i.e., a more even mix, and higher CPRs tend to go together. When all countries (latest surveys) are divided in half by low vs. high AD values (above and below the median), the average CPR value is 48.9% for low-AD countries, with less skew, and only 41.0% for the high-AD countries, with more skew. This holds true within both the low and the high range of contraceptive use. Within the low range (below the median) of CPRs, countries with high ADs average a CPR of only 19.6%, compared with a 23.7% CPR for countries with low ADs. Similarly, for countries in the high range of CPRs, the figures are 58.9% vs. 62.4%, respectively.

These patterns are consistent with the likelihood that broadening the method mix can help lead to a higher overall level of contraceptive use, as found for 64 countries cross-classified by the variability of access among methods and the overall level of access.^17^ The highest CPRs were found where average access was both high and relatively even among methods.

#### Method Gains and Losses

The way by which the method mix changes takes many forms, and the interplay differs considerably for which methods gain and which ones lose. Table 4 summarizes the shifts in the 15 countries with marked changes to their method mixes over time, arranged by region. In most countries, women have been moving from traditional to modern contraceptive methods. The other notable trend is that the injectable, as a newer method, shows only gains, never a loss. In some countries, condoms have risen in the mix (and not necessarily in settings with high HIV/AIDS prevalence). In a few countries where one method was extremely dominant over the years, that method has lost some ground recently. In other countries, rather irregular shifts have occurred, following different paths, but the net effect has still been a steady decline in the AD values. Variability among the countries is the overriding tendency, as was evident also among regions.

**Table 4.** Shifts in Contraceptive Method Mix by Country and Region for 15 Countries

**Region/Country**	**Trends in the Method Mix**
**sub-Saharan Africa**	
Benin	The injectable and pill have risen, while sterilization, the IUD, the condom, and especially traditional methods have fallen.
Mali	The injectable has risen substantially with declines in the pill and traditional methods.
Rwanda	Since the disruptions of the mid-1990s, the injectable has risen to over half of all use, while traditional methods have declined correspondingly. The implant gained in the latest survey.
Uganda	The injectable has risen at the expense of traditional methods, with a recent increase by the implant.
**North Africa & West Asia**	
Egypt	The IUD rose quite remarkably to a high level, with a corresponding decline for the pill. Recently, the injectable has shown some increase.
Iran	Sterilization has risen steadily; in the last survey, it lost ground to a resurgence in the pill, while traditional methods lost ground.
Turkey	The extensive use of traditional methods gave way to a rise in the IUD and condom, as well as female sterilization.
**Asia**	
Mongolia	The pill, injectable, and condom have risen while the IUD and traditional methods have fallen.
Viet Nam	The historic dominance of the IUD has weakened as shares of the pill and condom have gained.
**Latin America**	
Colombia	Sterilization rose very sharply over the years, along with a small rise for condoms. Shares for the pill, IUD, and traditional methods declined.
Guyana	The pill and traditional methods have lost ground, while the other methods show irregular trends that balance out to reduce skew.
Haiti	The picture changed sharply from 1994/95 onward. Sterilization declined while the injectable rose, with irregularities for other methods.
Honduras	The injectable share increased sharply with declines in the pill and in traditional methods.
Paraguay	Shares of the injectable and condom are up while shares of the IUD and traditional methods have fallen.
Peru	The IUD is down, as is sterilization slightly, while the injectable and especially the condom have risen.

#### Patterns of Movement Toward a More Balanced Method Mix

Notwithstanding the variability, we can take note of certain patterns, recognizing that these must be somewhat approximate. Four tendencies emerge, each of which is illustrated below in a chart for one of the countries to clarify how each mix developed over time. Because the method mix is often confused with the CPR level, the x-axis includes the CPR for each year so any point on the curve can be translated to the percentage of all married women using the method.

**Figure 5.**
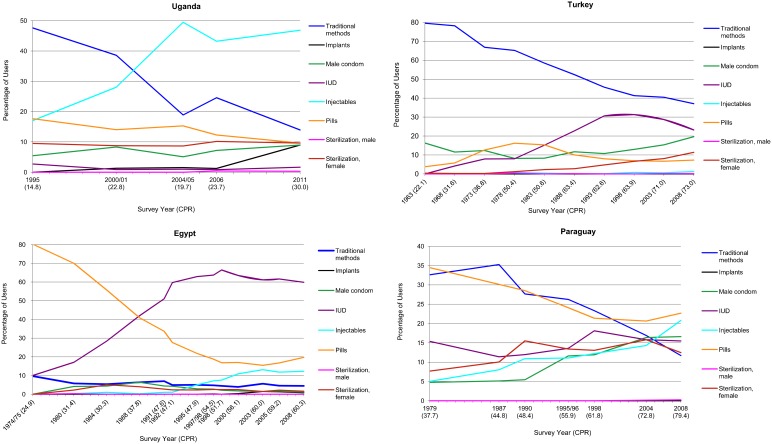
Changes in the Method Mix Over Time, 4 Illustrative Countries Abbreviation: CPR, contraceptive prevalence rate; IUD, intrauterine device.

**Takeoff of one method partially offset by changes among other methods.** The first pattern, found in 7 countries (Benin, Haiti, Honduras, Mali, Peru, Rwanda, and Uganda) is a marked rise by one contraceptive method that is partially offset by changes among the other methods. The one method starts well below the average use of 12.5%, which would be its share if women were using all 8 contraceptive methods equally, and rises well above it; the overall result is less total dispersion in the shares among the methods than before, and a reduced AD value. The rise of the injectable is the primary example, illustrated by Uganda (Figure 5), where the rise in the injectable share is nearly a mirror image of the decline in the share of traditional methods. The pill has lost ground while the implant is up in the latest survey. The other methods have been flat, and male sterilization has a trivial share, as it does elsewhere.

**Systematic replacement of traditional methods with other methods.** A second pattern is the systematic replacement of traditional method dominance by the IUD and condom. Turkey is an outstanding example; it was known for many years as the example of fairly high contraceptive prevalence based just on traditional methods, primarily withdrawal (Figure 5). The surveys, however, show a marked decline in withdrawal as modern methods replaced it, especially the IUD, with recent gains by the condom and female sterilization. Colombia is another example of nearly all methods having shares close together, except for female sterilization, the most-used method, whose high level reflects an accumulation over many years.

**Continued but declining domination by a single method.** A third pattern shows a single method that is both dominant and stable, with minor losses recently. This pattern is illustrated by Egypt and Viet Nam. In Egypt, the pill was the primary method at the start, with all other methods at low levels (Figure 5). However, it lost ground sharply in favor of the IUD, which rose to a stable level of three-fifths of all contraceptive use in the country. The pill plateaued at 15%–20%, while the injectable had a recent rise. The other methods are still minimal, including condoms and even traditional methods, which interestingly were never popular in Egypt. Over the 33 years shown, the CPR has risen from 25% to 60%.

**Declines in dominant methods with increases in other methods toward a balanced method mix.** A fourth pattern, illustrated by Paraguay, shows movement from marked distortion to an unusually balanced mix (Figure 5); it has the lowest AD value of 6.4 among all the countries. At the beginning of the survey period, the pill and traditional methods were highest, but by the end, all methods had similar shares except for male sterilization and the implant. The CPR in Paraguay rose from 38% in 1979 to 79% by 2008, illustrating a case of improved choice along with widespread use of all methods.

Paraguay shows general movement from marked distortion to an unusually balanced method mix.

**Mixed patterns.** Besides the countries mentioned above, the remaining 3 of the 15 selected countries, Guyana, Iran, and Mongolia, show somewhat irregular trends that involve different sets of methods.

## DISCUSSION

Previous measurements of distortions in contraceptive method mix have used the 50% rule while our new measure uses the average deviation of method shares around their own mean. Using national survey data from 123 countries, our analysis finds that both rules are useful. The 50% rule detects whether a country stands out as having a distortion in its method mix when a single method accounts for over half of all contraceptive use, while the AD rule is sensitive to the net change across all methods, without necessarily showing whether any one method is very dominant. Each approach has limitations covered by the other one; the 50% rule does not provide the pattern for the non-dominant methods and the AD rule does not tell whether a single method takes an extreme value.

Both the 50% rule and the average deviation rule are useful measures of method mix distortion.

In many countries, the method mix is firmly entrenched, showing little movement for a long time. However, the possibility of change can never be discounted, given the historic surprises of the emergence of the injectable in Indonesia and South Africa and then in much of eastern and southern Africa. The pill may see increased use in the future, as it is acceptable and present in a wide variety of countries. The IUD, so popular in the Middle East, has never been used extensively in sub-Saharan Africa, but a myriad of pilot projects over the years for various methods have shown that intensive efforts can elicit impressive increases in the use of neglected methods.^18^^,^^19^ In both sub-Saharan Africa and the Middle East, sterilization is minimal; for such a clinical method to increase its share in the method mix, a great deal of infrastructure work and training in the private or public sector is clearly necessary—a challenge in the poorest countries. However, the implant has shown small but definite gains in some African countries and will likely gain more users given increasing donor support for the method.^20^ Male sterilization has not won a wide following in Latin America, the Middle East, or much of Asia; also where the CPRs are already quite high, they may signal relatively little potential for a change in method mix.

The prospects for favorable changes will almost certainly involve different paths in the various settings, as they have in the past. In some, 1 or 2 methods will account for most change, and that can either improve the balance among methods or, while raising the CPR substantially, distort the method mix from its current state. In the longer run, new methods may emerge that gain popularity among users. In future surveys, repeated application of both the 50% rule and the AD measure can strengthen the monitoring of these developments and extend the time series, at very little effort.

Historically, it is likely that in the early days of experience with modern contraceptives, traditional methods held center stage in the method mix since they were the only ones known in the culture, including some abstinence and deliberate extensions of breastfeeding. As modern methods became better known and total contraceptive prevalence rose, the method mix moved toward a better spread among methods but in quite different ways. In each country, the public was experimenting and gaining experience with alternative choices. At the same time, a net reduction in unmet need for family planning occurred over time in many countries, as the rise in contraceptive use outpaced declines in the desired family size.

Regarding total contraceptive prevalence, few countries have CPRs in the seventies without a substantial share of sterilization in the mix, and even those countries with extensive IUD use have generally risen to CPRs only in the mid-sixties. In developing countries, total fertility rates are usually at replacement level only in those countries with considerable use of sterilization. Interesting exceptions are Viet Nam and Turkey, which rely on a combination of the IUD and traditional methods, with extensive use of abortion in Viet Nam and perhaps in Turkey as well.^21^ Use of short-acting methods comes with high discontinuation rates, constraining total contraceptive use and the fertility effects.

Few countries have high contraceptive prevalence rates without a substantial share of sterilization in the mix.

How can both the method mix and the total contraceptive prevalence be improved? When we say that the method mix should be improved, we are really saying that some of the less-favored methods should be made more available and attractive and that promising new methods should be introduced. This would meet the needs of more women and couples, it would put the mix into better balance, and it would raise overall contraceptive use. However, there are questions of strategy. Apart from the usual counsel to “try harder” to improve accessibility and quality, with better training and supervision, and an expansion of services to the private sector, efforts to improve the method mix would focus on making the neglected methods in each country more available and on introducing new methods where feasible. However, the CPR itself may rise faster under a different strategy, posing the question of whether better implementation of the popular methods may prove more cost-effective than efforts to advance methods that so far have won little response. Where the public in a country clearly dislikes a method and the national authorities neglect it, as with male sterilization in most countries, the condom in many, and the IUD in some, it may be a better strategy to improve access to and quality for the established methods, both to enhance actual choice and to raise the CPR level. The answer to such questions will vary; sometimes a relatively new method shows promise, as the injectable clearly did and as the implant currently does in some countries. That points to a policy favoring a combination of improved implementation of established methods together with attention to neglected ones and to promising new ones. Whatever the strategy, it should embrace measures to advance free and informed choice.

Both the mix and the CPR may be improved through at least 2 basic approaches. The first is for special attention to go to the large market of discontinuers from resupply methods, both to prolong use for some users^22^ and to offer ready alternatives for others. Very large numbers of injectable users are discontinuing use throughout eastern and southern Africa, by simply not showing up for the next shot. With proper counseling at the point-of-service, they can be encouraged toward a longer trial of the method or toward adopting alternative methods promptly after discontinuing their current method.

The second approach to improving both the mix and the CPR is the strategic focus on postabortion and postpartum women. The leverage in that focus is not generally recognized; it automatically separates out the relevant segment of the whole population as it moves through its next pregnancy experience. Apart from first births, most women who will ever have a next pregnancy or birth will do so within the next 5 years, and during that period many or most will encounter the various services related to pregnancy, birth, and child care. A more determined and disciplined focus on those programs would in many settings enlarge choice, meet women's needs faster, improve the balance of methods in the mix, and raise the CPR.

Survey findings of the kind reported here need to be augmented by local studies to investigate reasons why certain methods experience a takeoff. Central program initiatives always interact with public preferences and private-sector initiatives. Lessons from such studies can be drawn from cases like Uganda's, where the injectable has replaced traditional methods and where other methods, including the implant, now claim larger shares. A different type is that of Egypt, in which the IUD has proven popular, as it has elsewhere in the Middle East. Egypt illustrates the case of a strong private medical sector, which may also have had a role in the recent increase of the injectable. In neither region has sterilization been substantial. In sub-Saharan Africa the IUD has also been quite minor, and the availability of long-acting methods remains very problematic.

Supply and demand factors drive both method availability and method use. Such factors include policy and programmatic changes undertaken by the public sector, but they can also include widespread changes in social norms, economies, and the growing availability (and popularity) of private-sector health care services. A more detailed historical analysis of the conditions surrounding these changes can be instructive to enhance the understanding of what mediates these transitions and the extent to which they are replicable in other countries.

## Notes

**Peer Reviewed**

**First published online:** 2015 Feb 25

## Appendix

**APPENDIX 1.** 123 Countries Included in the “Average Deviation” Method Mix Analysis, by Region

**Country**	**Latest Survey Year**
**sub-Saharan Africa**	
Angola	2001
Benin	2006
Burkina Faso	2010/11
Burundi	2010/11
Cameroon	2011
Cape Verde	2005
Central African Republic	2006
Chad	2004
Comoros	2000
Congo	2011/12
Congo, Democratic Republic of	2010
Côte d'Ivoire	2006
Djibouti	2006
Equatorial Guinea	2000
Eritrea	2002
Ethiopia	2010/11
Gabon	2000
Gambia	2001
Ghana	2008
Guinea	2005
Guinea-Bissau	2006
Kenya	2008/09
Lesotho	2009/10
Liberia	2007
Madagascar	2008/09
Malawi	2010
Mali	2006
Mauritania	2007
Mauritius	2002
Mozambique	2011
Namibia	2006/07
Niger	2006
Nigeria	2011
Rwanda	2010/11
Sao Tome and Principe	2008/09
Senegal	2010/11
Sierra Leone	2008
Somalia	2006
South Africa	2003/04
South Sudan	2006
Sudan	2010
Swaziland	2010
Tanzania	2009/10
Togo	2006
Uganda	2011
Zambia	2007
Zimbabwe	2010/11
**North Africa & West Asia**	
Algeria	2006
Armenia	2010
Azerbaijan	2006
Bahrain	1995
Egypt	2008
Georgia	2005
Iran	2002
Iraq	2011
Jordan	2009
Kuwait	1999
Lebanon	1996
Libya	1995
Morocco	2003/04
Oman	2000
Qatar	1998
Saudi Arabia	1996
Syria	2006
Tunisia	2006
Turkey	2008
United Arab Emirates	1995
Yemen	2006
**Latin America**	
Argentina	2004/05
Belize	2006
Bolivia	2008
Brazil	2006
Chile	2006
Colombia	2010
Costa Rica	2010
Cuba	2006
Dominican Republic	2007
Ecuador	2004
El Salvador	2008
Guatemala	2002
Guyana	2009
Haiti	2005/06
Honduras	2005/06
Jamaica	2002/03
Mexico	2006
Nicaragua	2006/07
Panama	2009
Paraguay	2008
Peru	2010
Puerto Rico	2002
Suriname	2006
Trinidad and Tobago	2006
Uruguay	2004
Venezuela	1998
**Central Asia**	1998
Kazakhstan	2006
Kyrgyzstan	2005/06
Tajikistan	2007
Turkmenistan	2000
Uzbekistan	2006
**Asia & Pacific**	
Afghanistan	2010
Bangladesh	2011/12
Bhutan	2010
Cambodia	2010/11
China	2006
Hong Kong	2007
India	2007/08
Indonesia	2007
Korea, Republic of	2009
Lao People's Democratic Republic	2005
Malaysia	2004
Maldives	2009
Mongolia	2008
Myanmar	2009/10
Nepal	2011
Pakistan	2012/13
Papua New Guinea	1996
Philippines	2011
Singapore	1997
Solomon Islands	2006/07
Sri Lanka	2006/07
Thailand	2009
Timor-Leste	2009/10
Viet Nam	2010/11

**APPENDIX 2.** Method Mix Among Contraceptive Users: Improvements in 15 Countries

**Country**	**Survey Year**	**Sterilization, Female**	**Sterilization, Male**	**Pills**	**Injectables**	**IUD**	**Condom**	**Implants**	**Traditional Methods**	**Average Deviation Value**
**sub-Saharan Africa**										
Benin	1981/82	-	1.5	4.5	-	1.5	3.0	-	89.4	19.2
Benin	1996	2.5	-	6.1	4.3	3.1	4.3	-	79.8	16.8
Benin	2001	1.6	-	9.8	11.4	4.3	7.1	1.6	64.1	12.9
Benin	2006	1.8	-	8.9	10.7	3.6	6.5	3.0	65.7	13.3
Mali	1987	2.2	-	19.6	2.2	2.2	-	-	73.9	17.1
Mali	1995/96	4.6	-	47.7	3.1	4.6	6.2	-	33.8	14.1
Mali	2001	3.7	-	34.6	25.9	2.5	3.7	1.2	28.4	12.8
Mali	2006	3.7	-	35.4	30.5	1.2	4.9	1.2	23.2	12.9
Rwanda	1983	-	-	1.8	3.6	2.7	-	-	91.8	19.8
Rwanda	1992	3.3	-	14.2	39.8	0.9	0.9	1.4	39.3	14.0
Rwanda	1996	-	-	17.4	29.7	2.2	1.4	1.4	47.8	14.4
Rwanda	2000	6.0	-	7.5	14.3	2.3	3.0	-	66.9	14.1
Rwanda	2005	2.9	-	13.9	27.2	1.7	5.2	-	49.1	13.2
Rwanda	2007/08	1.9	0.3	17.6	41.8	0.5	5.2	4.4	28.3	12.5
Rwanda	2010/11	1.6	-	13.8	51.1	1.0	5.6	12.2	14.8	10.5
Uganda	1988/89	16.3	-	22.4	8.2	4.1	-	-	49.0	12.6
Uganda	1995	9.5	-	17.7	17.0	2.7	5.4	-	47.6	11.2
Uganda	2000/01	8.8	-	14.0	28.1	0.9	8.3	1.3	38.6	10.8
Uganda	2004/05	8.7	-	15.3	49.5	1.0	5.1	1.5	18.9	11.5
Uganda	2006	10.2	0.4	12.3	43.2	0.8	7.2	1.3	24.6	10.7
Uganda	2011	9.6	0.3	9.6	46.8	1.7	9.0	9.0	14.0	8.9
**North Africa & West Asia**										
Egypt	1974/75	-	-	80.2	-	10.1	-	-	9.7	16.9
Egypt	1980	2.3	0.3	70.0	0.3	17.1	4.2	-	5.8	15.5
Egypt	1984	5.1	-	55.7	1.0	28.4	4.4	-	5.4	14.8
Egypt	1988	4.0	-	40.9	0.3	42.0	6.4	-	6.4	14.5
Egypt	1991	2.8	-	33.7	1.1	51.1	4.4	-	7.0	14.9
Egypt	1992	2.4	-	27.6	1.1	59.7	4.3	-	4.9	15.6
Egypt	1995	2.3	-	21.8	5.0	62.9	2.9	-	5.0	14.9
Egypt	1997/98	2.6	-	18.8	7.2	63.7	2.8	0.2	4.8	14.4
Egypt	1998	2.5	-	16.9	7.6	66.5	2.1	-	4.5	14.6
Egypt	2000	2.5	-	17.0	10.9	63.5	1.8	0.4	3.9	13.9
Egypt	2003	1.5	-	15.5	13.2	61.2	1.5	1.5	5.7	13.1
Egypt	2005	2.2	-	16.7	11.8	61.7	1.7	1.4	4.6	13.3
Egypt	2008	1.7	-	19.7	12.3	59.9	1.2	0.8	4.5	13.7
Iran	1976/77	5.7	-	49.7	-	4.0	11.5	-	29.0	13.4
Iran	1989	8.0	-	36.3	-	7.4	11.4	-	36.9	12.0
Iran	1992	11.8	1.4	35.0	-	11.0	9.9	-	31.0	10.2
Iran	1993	13.7	1.5	36.5	-	10.7	10.0	-	27.7	10.1
Iran	1994	16.3	1.8	32.3	0.7	11.5	9.7	-	27.8	9.7
Iran	1995	19.3	1.8	32.1	1.8	10.0	8.0	-	27.0	10.2
Iran	1996	20.6	2.2	30.0	3.4	11.4	7.7	-	24.7	9.5
Iran	1997	21.4	2.6	28.9	4.0	11.5	7.5	0.7	23.4	9.1
Iran	2000	23.2	3.7	25.0	3.8	11.5	8.0	0.7	24.2	8.7
Iran	2002	20.8	3.1	34.3	3.1	10.4	8.2	0.5	19.5	9.3
Turkey	1963	0.4	-	3.8	-	-	16.3	-	79.5	17.7
Turkey	1968	0.3	-	5.8	-	4.2	11.5	-	78.2	16.4
Turkey	1973	0.3	-	12.6	-	7.9	12.4	-	66.8	13.6
Turkey	1978	1.2	0.4	16.2	0.8	8.0	8.2	-	65.2	14.1
Turkey	1983	2.3	-	15.4	0.4	15.1	8.3	-	58.5	12.9
Turkey	1988	2.8	0.2	10.1	0.2	22.7	11.7	-	52.4	12.5
Turkey	1993	4.7	-	8.0	0.2	30.6	10.7	-	45.8	12.8
Turkey	1998	6.6	-	7.0	0.8	31.3	13.0	-	41.3	12.0
Turkey	2003	8.1	0.1	6.7	0.6	28.7	15.3	-	40.5	11.8
Turkey	2008	11.4	0.1	7.3	1.2	23.2	19.6	-	37.1	10.6
**Latin America**										
Colombia	1969	-	-	23.4	-	13.2	7.3	-	56.1	13.8
Colombia	1976	10.0	0.5	33.2	1.0	21.2	4.2	-	29.9	11.7
Colombia	1978	17.2	-	39.5	2.3	17.7	3.2	-	20.0	11.1
Colombia	1980	22.5	0.4	36.6	4.2	17.1	3.2	-	16.0	10.6
Colombia	1986	29.3	0.6	26.2	3.8	17.6	2.7	-	19.7	10.7
Colombia	1990	32.5	0.8	21.9	3.4	19.3	4.5	-	17.7	10.3
Colombia	1995	36.3	1.0	18.2	3.5	15.7	6.1	1.0	18.2	9.6
Colombia	2000	35.5	1.3	15.5	5.2	16.3	8.0	0.3	18.0	8.8
Colombia	2004/05	40.2	2.3	12.5	7.5	14.4	9.1	0.4	13.6	7.7
Colombia	2010	44.2	4.3	9.6	11.6	9.5	8.9	3.9	8.0	7.9
Guyana	1975	27.9	-	29.5	-	18.4	-	-	24.3	12.5
Guyana	1991/92	17.3	-	35.1	5.2	24.1	14.8	-	3.6	10.3
Guyana	2000	12.2	0.3	30.4	10.0	17.1	23.8	2.7	3.5	8.4
Guyana	2005	8.8	-	36.0	11.2	22.4	18.0	0.3	3.2	9.7
Guyana	2006	5.1	-	38.1	10.7	16.4	17.3	8.0	4.5	8.6
Guyana	2009	12.5	-	21.7	11.3	17.2	30.4	0.5	6.4	8.0
Haiti	1977	1.1	1.1	17.4	1.1	2.6	5.8	-	71.1	15.9
Haiti	1983	10.1	1.4	31.9	2.9	2.9	7.2	-	43.5	12.6
Haiti	1987	18.8	-	33.3	11.6	5.8	2.9	-	27.5	10.6
Haiti	1989	24.8	-	40.6	15.8	5.9	5.0	-	7.9	10.9
Haiti	1994/95	17.1	-	17.1	14.9	3.3	14.4	6.6	26.5	6.9
Haiti	2000	9.9	-	8.2	41.8	2.1	10.3	7.1	20.6	9.4
Haiti	2005/06	6.5	-	10.2	34.1	1.9	16.4	5.0	26.0	9.7
Honduras	1981	30.5	0.8	44.7	1.1	9.2	1.1	-	12.6	12.6
Honduras	1984	35.0	0.6	36.7	0.9	11.0	2.6	-	13.3	11.9
Honduras	1987	31.3	0.5	33.3	0.7	10.7	4.5	-	18.9	11.5
Honduras	1991/92	33.6	0.4	21.8	1.1	11.0	6.3	-	25.9	10.9
Honduras	1996	36.3	0.2	19.8	2.2	17.0	6.4	-	18.0	10.3
Honduras	2001	29.1	0.3	16.8	15.5	15.5	5.2	-	17.5	8.0
Honduras	2005/06	32.5	0.5	17.3	21.2	10.1	4.4	-	14.0	8.7
Paraguay	1979	7.7	-	34.5	5.0	15.4	4.8	-	32.6	11.2
Paraguay	1987	10.0	-	30.1	8.0	11.4	5.1	-	35.3	10.1
Paraguay	1990	15.5	-	28.5	10.9	11.9	5.5	-	27.7	8.5
Paraguay	1995/96	13.4	-	24.1	11.1	13.6	11.6	-	26.3	6.8
Paraguay	1998	13.1	-	21.4	12.2	18.1	11.9	-	23.3	6.5
Paraguay	2004	15.8	0.1	20.6	14.3	15.8	16.4	-	16.9	6.2
Paraguay	2008	12.5	0.3	22.7	20.8	15.5	16.6	-	11.7	6.4
Peru	1969/70	8.0	-	12.0	-	4.0	12.0	-	64.0	12.9
Peru	1977/78	9.2	-	13.7	3.3	4.2	3.6	-	66.0	13.7
Peru	1981	10.0	-	12.5	5.0	10.0	2.5	-	60.0	11.9
Peru	1986	13.6	-	14.5	2.9	16.5	1.6	-	50.9	11.4
Peru	1991/92	13.6	0.2	9.8	3.3	23.1	4.8	-	45.2	11.1
Peru	1996	15.0	0.3	9.8	12.6	18.9	6.9	0.5	36.1	8.1
Peru	2000	18.0	0.7	9.8	21.7	13.3	8.2	0.3	28.0	7.7
Peru	2004/06	14.6	0.6	10.1	20.7	7.9	11.9	-	34.3	8.0
Peru	2007/08	13.6	0.4	10.6	22.8	5.6	13.8	-	33.2	8.4
Peru	2009	12.9	0.6	10.5	24.9	5.2	13.8	-	32.1	8.4
Peru	2010	12.5	0.5	11.2	23.6	4.4	15.1	0.1	32.6	8.4
**Asia**										
Mongolia	1994	1.6	-	4.4	1.9	57.7	5.9	0.5	28.0	15.2
Mongolia	1998	4.0	-	7.0	5.2	53.8	5.8	0.3	23.9	13.2
Mongolia	2000	1.9	0.3	12.4	8.7	50.2	6.4	0.4	19.5	11.2
Mongolia	2003	4.4	-	16.0	8.4	47.6	7.8	0.4	15.4	10.4
Mongolia	2005	3.7	0.2	17.6	16.9	44.1	8.1	1.2	8.2	10.3
Mongolia	2008	4.7	-	17.7	14.4	40.6	12.2	0.4	10.0	8.8
Viet Nam	1988	5.1	0.6	0.8	0.4	62.1	2.3	-	28.9	16.5
Viet Nam	1994	6.0	0.3	3.2	0.3	51.3	6.2	-	32.7	14.7
Viet Nam	1997	8.4	0.7	5.7	0.3	51.2	7.8	-	25.9	13.0
Viet Nam	2000	8.2	0.8	7.3	0.5	49.9	8.2	-	25.0	12.5
Viet Nam	2000	7.9	0.6	9.5	0.7	57.4	8.0	-	16.0	12.1
Viet Nam	2001	7.7	0.5	10.0	0.7	55.8	7.9	-	17.3	12.0
Viet Nam	2002	7.5	0.6	8.0	0.5	48.0	7.4	-	27.9	12.7
Viet Nam	2002	7.2	0.5	10.4	0.9	56.6	8.5	-	15.9	11.9
Viet Nam	2003	6.9	0.5	11.4	0.9	57.0	7.4	-	15.7	11.9
Viet Nam	2004	6.6	0.4	11.9	1.1	56.0	9.3	-	14.7	11.4
Viet Nam	2005	6.3	0.4	12.5	1.2	55.5	9.7	-	14.5	11.3
Viet Nam	2006	5.5	0.2	10.6	1.5	54.9	11.0	-	16.3	11.6
Viet Nam	2006	7.7	0.7	11.9	1.6	47.6	10.1	0.1	20.3	10.7
Viet Nam	2007	5.6	0.4	13.2	1.1	55.4	10.5	0.1	13.7	11.2
Viet Nam	2010/11	5.0	0.1	13.0	2.2	40.0	16.4	0.3	23.0	10.6
